# Some Observations on the Prevailing Opinions on the Modus Operandi of the Causes Producing Compression of the Brain

**Published:** 1820-11

**Authors:** 


FOR THE LONDON MEDICAL AND PHYSICAL JOURNAL.

### *Some Observations on the prevailing Opinions on the Modus Operandi of the Causes producing Compression of the Brain.* By R. S.

[In continuation from p. 193.]

I HAVE to acknowledge the attention you have given my communication; and also thank you for the useful hints contained in your Journal for July last, under the head of "Notices to Correspondents," and addressed, of course, to me, whom you very rightly conclude to be a young student.

I would, however, here remark, that, although my view of the opinions of the parties may appear too exclusive, yet the lecturer whose opinions I particularly allude to, will be found, almost invariably, when drawing his conclusions upon the subject of my observations, as well as on some other affections of the nervous system, to attribute the various phenomena solely to the operation of mechanical causes on the circulation of blood in the substance of the brain. This, then, is overlooking entirely the primary effects which the same causes must produce on the nervous, independently of the vascular, system; for it will not be denied, that an unaccustomed and undue pressure on any part of the nervous system, must more or less derange the functions of the organ immediately connected with, or dependant upon, the natural state of the nerve compressed, without the said pressure being in such degree, or so applied, as to operate mechanically by impeding or suspending the circulation of blood in the nervous structure. We know that the vascular system is intimately connected with, and much dependant upon, nervous influence; and therefore, whatever change the pressure of a foreign body upon, or its simple contact with, the nervous matter of the brain, may produce on this nervous influence, by it must the vascular action be regulated. Therefore, to say that the symptoms occasioned by a compression of the brain arise from the diminished capacity for the vascular system, as the lecturer I have alluded to affirms, is to shut our eyes from the view of that wonderful, but intricate, subject—the nervous system, the laws of which may be said to be regulated by, and centre in, the functions of the brain.

Now, admitting that I have taken but an exclusive view of the opinions on this matter, it will be remembered that my object was merely to show the incongruities obtaining in the reasonings of some physiologists; and, to expose the grounds on which they rested, appeared to me best calculated to accomplish such a purpose: since, when the chief grounds of an argument are shown and examined, the merits of the conclusions drawn from them are seen from the bottom. And it is, in fact, to the propositions upon which the lecturer in question founds his arguments respecting the *modus operandi* of the causes producing the symptoms of compression of the brain, to which I particularly alluded; because I think them untenable, inasmuch as they are unsupported by facts. Indeed, his view of the matter leaves totally unaccounted for the same train of symptoms originating from causes whose very nature and mode of application demonstrably prove that they do not operate mechanically, by impeding or diminishing the circulation of blood in the brain or nervous structure, and yet give rise to the same symptoms as those which he asserts to be the effect of a diminished circulation. What proofs or probability exist in support of his conclusion, yet remain to be shown; for, if such causes do exert any influence on the circulation, it must be secondary, and depend on the effects primarily resulting from their application: but what this influence is, I will not pretend to explain.

